# Synergistic effects of combined cisplatin and *Clinacanthus nutans* extract on triple negative breast cancer cells

**DOI:** 10.1016/j.jtumed.2023.04.003

**Published:** 2023-04-15

**Authors:** Nur Fitriyani Afiqah Binti Abu Bakar, Zhin Leng Yeo, Faisal Hussin, Priya Madhavan, Vuanghao Lim, Khairunadwa Jemon, Praseetha Prabhakaran

**Affiliations:** a Department of Biosciences, Faculty of Science, Universiti Teknologi Malaysia, Skudai, Malaysia; b Department of Chemistry, Faculty of Science, Universiti Teknologi Malaysia, Skudai, Malaysia; c School of Medicine, Faculty of Health and Medical Sciences, Taylor's University, Subang Jaya, Malaysia; d Advanced Medical and Dental Institute, Universiti Sains Malaysia, Kepala Batas, Penang, Malaysia

**Keywords:** البرمجة الذاتية للخلايا, سيسبلاتين, عشبة الثعبان, التمايز, سرطان الثدي السلبي الثلاثي, Apoptosis, Cisplatin, *C. nutans*, Differentiation, Triple Negative Breast Cancer (TNBC)

## Abstract

**Objective:**

Triple negative breast cancer (TNBC) is the most invasive breast cancer subtype enriched with cancer stem cells. TNBCs do not express estrogen, progesterone, or human epidermal growth factor receptor 2 (HER2) receptors, making them difficult to be targeted by existing chemotherapy treatments. In this study, we attempted to identify the effects of combined cisplatin and *Clinacanthus nutans* treatment on MDA-MD-231 and MDA-MB-468 breast cancer cells, which represent TNBC subtypes.

**Methods:**

The phytochemical fingerprint of *C. nutans* ethanolic leaf extract was evaluated by LC–MS/MS analysis. We investigated the effects of cisplatin (0–15.23 μg/mL), *C. nutans* (0–50 μg/mL), and a combination of cisplatin (3.05 μg/mL) and *C. nutans* (0–50 μg/mL), on cell viability, proliferation, apoptosis, invasion, mRNA expression in cancer stem cells (CD49f, KLF4), and differentiation markers (TUBA1A, KRT18) in TNBC cells. In addition, we also studied the interaction between cisplatin and *C. nutans*.

**Results:**

Derivatives of fatty acids, carboxylic acid ester, and glycosides, were identified as the major bioactive compounds with potential anticancer properties in *C. nutans* leaf extract. Reductions in cell viability (0–78%) and proliferation (2–77%), as well as a synergistic anticancer effect, were identified in TNBC cells when treated with a combination of cisplatin and *C. nutans*. Furthermore, apoptotic induction via increased caspase-3/7 activity (MDA-MB-231: 2.73-fold; MDA-MB-468: 3.53-fold), and a reduction in cell invasion capacity to 36%, were detected in TNBC cells when compared to single cisplatin and *C. nutans* treatments. At the mRNA level, cisplatin and *C. nutans* differentially regulated specific genes that are responsible for proliferation and differentiation.

**Conclusion:**

Our findings demonstrate that the combination of cisplatin and *C. nutans* represents a potential treatment for TNBC.

## المخلص

**أهداف البحث:**

سرطان الثدي الثلاثي السلبي هو أكثر أنواع سرطان الثدي غزوية غنية بالخلايا الجذعية السرطانية. سرطان الثدي السلبي الثلاثي لا يعبر عن مستقبلات هرمون الاستروجين والبروجسترون وعامل نمو البشرة البشري 2 ، مما يجعل من الصعب استهدافها من خلال العلاجات الكيميائية الحالية. خضعت هذه الدراسة لتحديد آثار العلاج المركب سيسبلاتين- عشبة الثعبان على خلايا سرطان الثدي " م.د.أ- م.د 231" و "م.د.أ- م.ب 468" التي تمثل الأنواع الفرعية لسرطان الثدي السلبي الثلاثي.

**طرق البحث:**

تم تقييم البصمة الكيميائية النباتية لمستخلص أوراق عشبة الثعبان باستخدام تحليل الكروماتوغرافيا السائلة مع طيف الكتلة المزدوج. تأثيرات سيسبلاتين (0-15.23 ميكروغرام / مل)، عشبة الثعبان (0-50 ميكروغرام / مل) وسيسبلاتين مشترك (3.05 ميكروغرام / مل) . تم فحص عشبة الثعبان (0-50 ميكروغرام / مل) على حيوية الخلية، والتكاثر، والاستماتة، والغزو، وتعبير للحمض النووي الريبي المراسل للخلايا الجذعية السرطانية (سي دي 94 إف ، عامل كربل المشابه 4) وعلامات التمايز (تي يو بي إيه ١ إيه ، كي آر تي 18) في خلايا سرطان الثدي الثلاثي السلبي. بالإضافة إلى ذلك سيسبلاتين وتفاعل عشبة الثعبان.

**النتائج:**

تم التعرف على مشتقات الأحماض الدهنية وإسترات الحمض الكربوكسيلي والجليكوزيدات كمركبات حيوية رئيسية ذات خصائص مضادة للسرطان المحتملة في مستخلص أوراق عشبة الثعبان تم عرض انخفاضات في حيوية الخلايا (0-78٪) والتكاثر (2-77٪) بالإضافة إلى تأثير مضاد للسرطان التآزري على هذه الخلايا المصابة بسرطان الثدي السلبي الثلاثي عند العلاج المشترك بين سيسبلاتين و عشبة الثعبان علاوة على ذلك ، تم عرض إحداث البرمجة الذاتية للخلايا عبر زيادة نشاط الكاسبيز-3/7 وتقليل قدرة اجتياح الخلايا إلى 36٪ في خلايا سرطان الثدي السلبي الثلاثي مقارنة بعلاج سيسبلاتين وعشبة الثعبان المنفرد. على مستوى الحمض النووي الريبوزي المرسال، قام سيسبلاتين وعشبة الثعبان بتنظيم جينات محددة بشكل مختلف والتي تتحكم في التكاثر والتمايز.

**الاستنتاجات:**

تشير هذه التأثيرات إلى علاج محتمل لسرطان الثدي السلبي الثلاثي مع العلاج المركب سيسبلاتين-عشبة الثعبان.

## Introduction

Breast cancer is known for its heterogeneity and consists of numerous tumor cells ranging from stem cell-like cells to more differentiated cells that determine the fate of the disease.^1^ A recent study by Sung et al. reported that the GLOBOCAN 2020 ranked female breast cancer as the most significant diagnosed cancer with a total number of 2.3 million new cases (11.7%) and the fifth ranked form of cancer in terms of mortality.^2^ Triple negative breast cancer (TNBC) lacks three important hormone receptors (ER, PR and HER2) and is responsible for 20% of all cases of breast cancer.^3^, ^4^, ^5^, ^6^ Of the intrinsic subtypes of breast cancer, TNBC is considered the most lethal subtype due to its clinically aggressive behavior and the absence of targeted therapies.^3^^,^^7^

In addition, TNBCs are known to be enriched with functional cancer stem-like cells which exhibit a high migration pattern and express some specific breast cancer genes which make these cells the most invasive subtype of all breast cancers.^8^^,^^9^ In general, CSCs are able to self-renew and differentiate into various cell types, thus resembling the role of healthy stem cells.^8^^,^^9^ Thus, this CSC sub-population is associated with chronic effects in various cancers including TNBCs. At present, the chemotherapeutic drugs that are used for the treatment of TNBC are often associated with significant toxicity and severe side effects.^10^^,^^11^ In addition, the presence of CSCs in TNBC tumors is often associated with the development of chemotherapy resistance following chemotherapy thus complicating TNBC treatment^12^ and leading to a highly metastatic and recurrent condition. Therefore, patients with TNBC are often difficult to treat and experience poorer survival rates when compared to patients with other breast cancer types. In recent years, TNBCs have shown specific sensitivity towards cisplatin, the first metal based anti-cancer drug.^1^^,^^13^^,^^14^

Cisplatin is a metal-based anti-tumor drug that has been found to be effective in the treatment of various cancers.^14^ This is a well-known cytotoxic drug that is claimed to be capable of interfering with DNA activity upon entering the nucleus of cells, thus preventing the DNA repair process and ultimately leading to cell death.^15^ Nevertheless, recent studies have shown that cisplatin may exhibit other mechanisms of action, apart from apoptosis, such as inducing the differentiation of cancer cells.^1^ Although previous studies reported that cisplatin can give rise to certain side effects following initial treatment,^16^^,^^17^ accumulating evidence shows that the combination of cisplatin with other potential anticancer drugs can induce either autophagy or apoptosis in various types of cancer cells.^14^^,^^17^^,^^18^ Cisplatin is also highly toxic to cancer cells with metastatic characteristics.^15^

The *Clinacanthus nutans* (*C. nutans*) plant has emerged as an important traditional herb that represents a potential chemoprevention alternative for cancer patients.^19^ *C. nutans* extracts contain various phytochemical compounds, including fatty acids, phenolics, glycosides, glycoglycerolipids, cerebrosides, and monoacylmonogalactosylglycerol, with useful biological capabilities.^20^, ^21^, ^22^, ^23^, ^24^, ^25^, ^26^ Naturally-derived phytochemical constituents in *C. nutans* extracts also exhibit cytotoxicity effects through the induction of apoptosis^23^ and antioxidant activity and could reduce the risk of cancer development.^20^^,^^24^^,^^25^ Furthermore, *C. nutans* is preferred by individuals because it is a natural herb that is relatively safe with fewer side effects than conventional drugs.^26^^,^^27^ In this study, we demonstrate the potential anticancer effects of a combination of cisplatin and *C. nutans* on MDA-MB-231 and MDA-MB-468 cells at the cellular and molecular levels.

## Materials and Methods

### Cell culture

MDA-MB-231 and MDA-MB-468 cell lines (Table 1) were purchased from the ATCC (American Type Culture Collection). Cells were cultured in DMEM/F12 + glu-tamax™ (Gibco by Life Technologies) containing 10% fetal bovine serum (FBS) (Thermo Fisher Scientific, USA), and 1% antibiotic (100 U/mL penicillin, 100 μg streptomycin/0.25 μg/mL) (Gibco by Life Technologies). The cells were maintained in T25 flasks (SPL Lifesciences, Korea) at 37 °C and 5% CO_2_. The cells were passaged approximately twice a week. The MDA-MB-231 and MDA-MB-468 cells used in experiments were derived from passage numbers 5–10 (P5–P10) to prevent significant variation between experiments.

**Table 1:** The triple negative breast cancer cell lines used in this study.^1^^,^^28^

Cell line	Tumor cell type	Tumor cell classification	Differentiation state	Prognosis
MDA-MB-231	Adeno-carcinoma	Claudin-low	Least differentiated and more stemness; more mesenchymal-like appearance	Worse prognosis
MDA-MB-468	Adeno-carcinoma	Basal-like	Differentiated; core basal-like	Moderately better prognosis

### *C. nutans* bioactive compound identification and extract preparation

The *C. nutans* ethanolic leaf extract was prepared in the Integrative Medicine Laboratory at the Advanced Medical and Dental Institute, Universiti Sains Malaysia (USM), Malaysia. The extract was subjected to various quality controls, as reported earlier.^29^^,^^30^ The plant was authenticated by its voucher specimen number 11465, and placed at the Herbarium Unit, School of Biological Sciences, USM, Malaysia.^29^^,^^30^ The leaves were first sorted, dried, and pulverized. The powder (255 g) was then macerated in 1L of ethanol in a conical flask at room temperature for three days with frequent stirring with a magnetic stirrer. The mixture was then filtered, and fresh ethanol was added to the residue. This procedure was performed several times until a clear and colorless solution was achieved. The filtrates were then mixed and evaporated under reduced pressure at 40 °C using a rotary evaporator (BUCHI Rotavapor R-200, Switzerland). Following ethanol evaporation, the dried extract was lyophilized at −50 °C in a freeze drier (CHRIST Alpha 1–4 LD plus Freeze Dryer, Germany) to remove all traces of moisture. The phytochemical fingerprint of *C. nutans* leaf extract was evaluated using LC–MS/MS analysis and a dual ESI source for both positive and negative ion mode analysis. The experiments were conducted to identify the presence and percentage of bioactive compounds in the extract. This was determined by the intensity arriving from the mass spectra of LC–MS/MS. To prepare the extract for treatment, a stock solution of aqueous extract was prepared by dissolving 100 mg of *C. nutans* extract in 1 mL of 10% Tween 20. Thus, the initial concentration of the aqueous stock solution was 100 mg/mL. The initial concentration of *C. nutans* was further diluted with serum-free media into several different concentrations (2.5, 5, 10, 20, 30, and 50 μg/mL) and further used to study anticancer effects on MDA-MB-231 and MDA-MB-468 breast cancer cells.

### Determination of cell viability and proliferation

A density of 3 × 10^4^ cells (100-μL/well) were seeded in flat bottom 96-well plates (Sarstedt, Newton, USA) and incubated for 24 h. Next, the MDA-MB-231 and MDA-MB-468 cells were treated with cisplatin (0, 0.76, 1.52, 3.05, 4.51, 6.10, and 15.23 μg/mL) (Tocris Bioscience, UK), *C. nutans* extract (0, 2.5, 5, 10, 20, 30 and 50 μg/μL) and a combination of cisplatin and *C. nutans* (3.05 μg/mL cisplatin + 0, 2.5, 5, 10, 20, 30 and 50 μg/μL), respectively. For the combined treatment, cells were treated with a fixed concentration of cisplatin (3.05 μg/μL) for 24 h prior to *C. nutans* (0, 2.5, 5, 10, 20, 30 and 50 μg/μL) treatment followed by further incubation at 24 h. A single concentration of cisplatin (3.05 μg/μL) was chosen and fixed in the combined treatment as this represented the main concentration where a significant drop in cell viability percentage was first observed. After 24h (single treatment) and 48 h (combined treatment) incubation, respectively, cell viability was assessed by a Cell Titer-Glo® 2.0 Assay (Promega, USA) according to the manufacturer's instructions. Cell proliferation was measured using the Cyquant NF Proliferation Assay (Invitrogen, Thermo Fisher Scientific) according to the manufacturer's instructions. Luminescence and fluorescence measurements were read using a GloMax®-Multi Detection System (Promega, USA). Experiments were performed in triplicate in three independent experiments. The IC_50_ concentration of each treatment was calculated using the Dose-Response-Special, X = log(concentration) formula in GraphPad Prism version 9 (GraphPad Software, Inc.).

### The interaction between cisplatin and *C. nutans*

The drug–drug interaction of the combined cisplatin and *C. nutans* treatment (fixed cisplatin concentration (3.05 μg/mL) followed by different concentrations of *C. nutans* (2.5, 5, 10, 20, 30 and 50 μg/mL)) was accomplished by conducting the isobologram-combination index analysis using CompuSyn software. The average inhibition effect, fa, of each treatment was calculated based on the median effect equation: *fa*/*fu* = (*D*/*Dm*) *m*. The inhibitory effect was determined based on cell viability data and the combination index (CI) for each treatment. The drug–drug interaction effect was demonstrated either as an antagonistic effect (CI value more than 1.0; additive effect: CI value = 1.0) or a synergistic effect (CI value less than 1.0).

### Detection of caspase-3/7 activity

Both MDA-MB-231 and MDA-MB-468 cells were treated with 20 nM Taxol (positive control) as well as IC_50_ concentrations of cisplatin, *C. nutans*, and a combination of cisplatin and *C. nutans*. Apoptotic activity was assessed via the Caspase-Glo® 3/7 Assay (Promega, USA) according to the manufacturer's instructions Luminescence measurements were read using a GloMax®-Multi Detection System (Promega, USA). Caspase-3/7 activity was expressed as the fold change in enzyme activity over untreated cells (control). All treatments were compared to untreated (control) cells, which were normalized to 1-fold of caspase-3/7 activity. The experiment was completed in triplicate to obtain average results.

### Detection of cell invasion

The MDA-MB-231 cells were treated with IC_50_ concentrations of cisplatin, *C. nutans*, a combination of cisplatin and *C. nutans*. The metastatic ability of MDA-MB-231 breast cancer cells upon cisplatin, *C. nutans* and combined cisplatin and *C. nutans* treatments using IC_50_ concentrations, were detected using the Cultrex® BME Cell Invasion Assay (Trevigen) in accordance with the manufacturer's instructions. The fluorescence intensity was read at 485 nm excitation and 520 nm emission using a GloMax®-Multi Microplate Multimode Reader (Promega, USA). The combined cisplatin and *C. nutans* treatment consisted of cells treated with cisplatin (3.05 μg/mL for 24 h) followed by *C. nutans* (10 μg/mL for 24 h). The invading cells were quantified based on metastatic index: (Metastatic index = (fluorescence reading (treatment))/(fluorescence reading (negative control)) × 100%). The experiment was completed in triplicates to obtain average results.

### Quantitative real time polymerase chain reaction (qRT-PCR)

MDA-MB-231 cells were lysed using the QIAzol® Lysis Reagent (Qiagen, Germany). Total cellular RNA was then extracted using an RNA Clean & Concentrator™-5 Kit (Zymo Research, USA). RNA quantity and quality were assessed with a NanoDrop 1000 (NanoDrop, Wilmington, DE, USA). Next, cDNA was synthesized from RNA using an iScript Synthesis Kit (Bio-rad, USA).

PCR reactions were then incubated in a Bio-rad iCycler TM Optical Module (Bio-rad, USA) at 42 °C for 30 min and 85 °C for 5 min to synthesize complementary DNA (cDNA) by reverse transcription. The final concentration of the cDNA product per sample was 20 ng/μL. Next, a mixture consisting of specific cDNA (1 μL), 2× SsoAdvanced™ universal SYBR® Green supermix (10 μL) (Bio-rad, USA) and nuclease-free water (9 μL) were added into a customized skirted 96-well PCR plate with specific targeted genes (Table 2) to detect the relative gene expression before and after drug treatment. Universal SYBR® Green supermix was used in the qRT-PCR assay to assist in the fluorescent signaling of gene expression levels.

**Table 2:** List of genes based on PrimePCR™ Assay validation.

Gene Name	UniGene ID	RefSeq accession number
Keratin 18 (*KRT18*)	Hs.406013	NC_000012.11, NG_008351.1, NT_029419.12
glyceraldehyde-3-phosphate dehydrogenase (*GAPDH*)	Hs.544577	NC_000012.11, NG_007073.2, NT_009759.16
integrin, alpha 6 (*ITGA6*)	Hs.133397	NC_000002.11, NT_005403.17, NG_008853.1
Kruppel-like factor 4 (gut) (*KLF4*)	Hs.376206	NC_000009.11, NT_008470.19
Tubulin, alpha 1A (*TUBA1A*)	Hs.654422	NC_000012.11, NG_008966.1, NT_029419.12

In addition, 1 μL of PCR control assay template was added into the positive control well. The PCR reactions were then incubated in a Bio-rad iCycler TM Optical Module (Bio-rad, USA) using a reaction protocol of 95 °C for 2 min (1 cycle for activation step), 95 °C for 5 s (40 cycles for denaturation step), 60 °C for 30 s (40 cycles for annealing/extension step), and 65–95 °C for 5 s (1 step for melt curve). Positive controls included PCR control assay templates (RQ1 and RQ2 (for RNA integrity)) and RT (for reverse transcriptase), while gDNA (genomic DNA contamination) was used as a negative control. Each PCR reaction was normalized to *GAPDH* (housekeeping gene) and plotted as relative mRNA expression. Experiments were performed in triplicate in two independent experiments. Bars represent mean ± SEM (n = 2). ∗p ≤ 0.05, ∗∗p ≤ 0.005, ∗∗∗p ≤ 0.0005, ∗∗∗∗p ≤ 0.0001.

### Statistical analysis

Analyses of the results were performed with Microsoft Excel and GraphPad Prism version 9 (GraphPad Software, Inc.). The Student's paired t-test with a two-tailed distribution was used to compare cisplatin, *C. nutans* and a combination of cisplatin and *C. nutans* and untreated MDA-MB-231 and MDA-MB-468 breast cancer cells. The results are presented as mean ± SEM. The significance is shown as: ∗p ≤ 0.05; ∗∗p ≤ 0.005; ∗∗∗p ≤ 0.0005; ∗∗∗∗p < 0.0001.

## Results

### Bioactive compounds of *C. nutans* with anticancer properties

The phytochemical fingerprint of *C. nutans* leaf extract was evaluated by LC–MS analysis using a dual ESI source for both positive and negative ion mode analysis. These experiments were conducted to determine the percentage of bioactive compounds present in the extract by determining the intensity derived from the mass spectra of LC–MS/MS (Table 3). The putatively identified compounds with positive ionization were homoarecoline (9.96%), C16 sphinganine (8.77%), emmotin A (16.86%), Val Trp Val (7.70%), palmitic amide (5.3%), oleamide (47.1%) and hydroxyhexanedecanoic acid (4.29%), while negative ionization mode revealed the presence of 2-C-methyl-D-erythritol-4-phosphate (4.06%), (E)-2,3-bis(ethoxycarbonylamino)but-2-enedioic acid (5.06%), idebenone (6.24%),(2*S*,3*R*)-2-[[2-[[(2*E*)-2-[(2,5-dimethoxyphenyl)methylidene]-3-oxo-1-benzofuran-6-yl]oxy]acetyl]xamino]-3-methylpentanoate (6.77%), 4-[[2-[3-(3,4-dihydro-2H-1,5-benzodioxepin-7-yl)-2-methyl-4-oxochromen-7-yl]oxyacetyl]amino]butanoate (12.31%), biotin-X-NHS (8.24%), dimethyl 2-(pentane-1-sulfonyl)butanedioate (18.93%), methyl 3-(5-O-carba-1-deoxy-alpha-d-glucopyranose-1-ylthio)-3-deoxy-alpha-d-mannopyranoside (13.17%), (6S)-dehydrovomifoliol (10.23%), and prostaglandin D2-d9 (4.68%) and 10-Methyl-9-[2-(propan-2-yl)phenoxycarbonyl] (10.29%).

**Table 3:** LC–MS/MS analysis of *C. nutans* leaf ethanolic extraction.

No.	Compound	Retention time	Molecular formula	Index	Mode	Peak percent (%)
1	2-C-Methyl-D-erythritol-4-phosphate	0.636	C_5_H_13_O_7_P	77.47	−	4.06
2	Homoarecoline	0.689	C_9_H_15_NO_2_	99.13	+	9.96
3	(E)-2,3-Bis(ethoxycarbonylamino) but-2-enedioic acid	1.042	C_10_H_14_N_2_O_8_	92.16	−	5.06
4	Idebenone	6.845	C_13_H_18_O_9_S	99.93	−	6.24
5	(2*S*,3*R*)-2-[[2-[[(2*E*)-2-[(2,5-dimethoxyphenyl)methylidene]-3-oxo-1-benzofuran-6-yl]oxy]acetyl]amino]-3-methylpentanoate	7.839	C_25_H_26_NO_8_	77.35	−	6.77
6	4-[[2-[3-(3,4-dihydro-2H-1,5-benzodioxepin-7-yl)-2-methyl-4-oxochromen-7-yl]oxyacetyl]amino]butanoate	8.558	C_25_H_24_NO_8_	76.83	−	12.31
7	Biotin-X-NHS	9.172	C_20_H_30_N_4_O_6_S	93.03	−	8.24
8	Dimethyl 2-(pentane-1-sulfonyl)butanedioate	9.475	C_11_H_20_O6_S_	99.92	−	18.93
9	Methyl 3-(5-O-carba-1-deoxy-alpha-d-glucopyranose-1-ylthio)-3-deoxy-alpha-d-mannopyranoside	9.909	C_14_H_26_O_9_S	99.25	−	13.17
10	C16 Sphinganine	12.223	C_16_H_35_NO_2_	99.75	+	8.77
11	Hydroxyhexane decanoic acid	12.371	C_16_H_32_O_3_	99.37	+	4.29
12	(6S)-dehydrovomifoliol	12.493	C_13_H_18_O_3_	99.39	−	10.23
13	Prostaglandin D2-d9	13.787	C_20_H_23_D_9_O_5_	96.65	−	4.68
14	Emmotin A	16.896	C_16_H_22_O_4_	98.66	+	16.86
15	Val Trp Val	17.965	C_21_H_30_N_4_O_4_	97.79	+	7.70
16	Palmitic amide	19.221	C_16_H_33_NO	99.92	+	5.30
17	Oleamide	19.487	C_18_H_35_NO	94.25	+	47.1
18	10-Methyl-9-[2-(propan-2-yl)phenoxycarbonyl]	20.271	C_24_H_22_NO_2_	89.79	−	10.29

A total of 18 major bioactive compounds with potential anticancer properties were identified by ethanol extraction.^31^, ^32^, ^33^, ^34^, ^35^, ^36^, ^37^, ^38^, ^39^ Of these, fatty acid derivatives (28.34%), carboxylic acid ester derivatives (26.68%), and glycoside derivatives (22.47%), were the major constituents of *C. nutans* leaf extract (Table 4).

**Table 4:** Percentage of bioactive compounds based on family of major constituents of *C. nutans* leaf extract.

Class	Percentage by class in major constituents of *C. nutans* leaf extract (%)
Alkaloid	4.98
Transferases	2.03
Amide	8.51
Fatty acid	28.34
Peptides	3.85
Glycoside	22.47
Carboxylic acid ester	26.68
Quinone	3.12

### The combination of cisplatin and *C. nutans* reduced the cell viability and proliferation of MDA-MD-231 and MDA-MB-468 breast cancer cells

**Figure 1:**
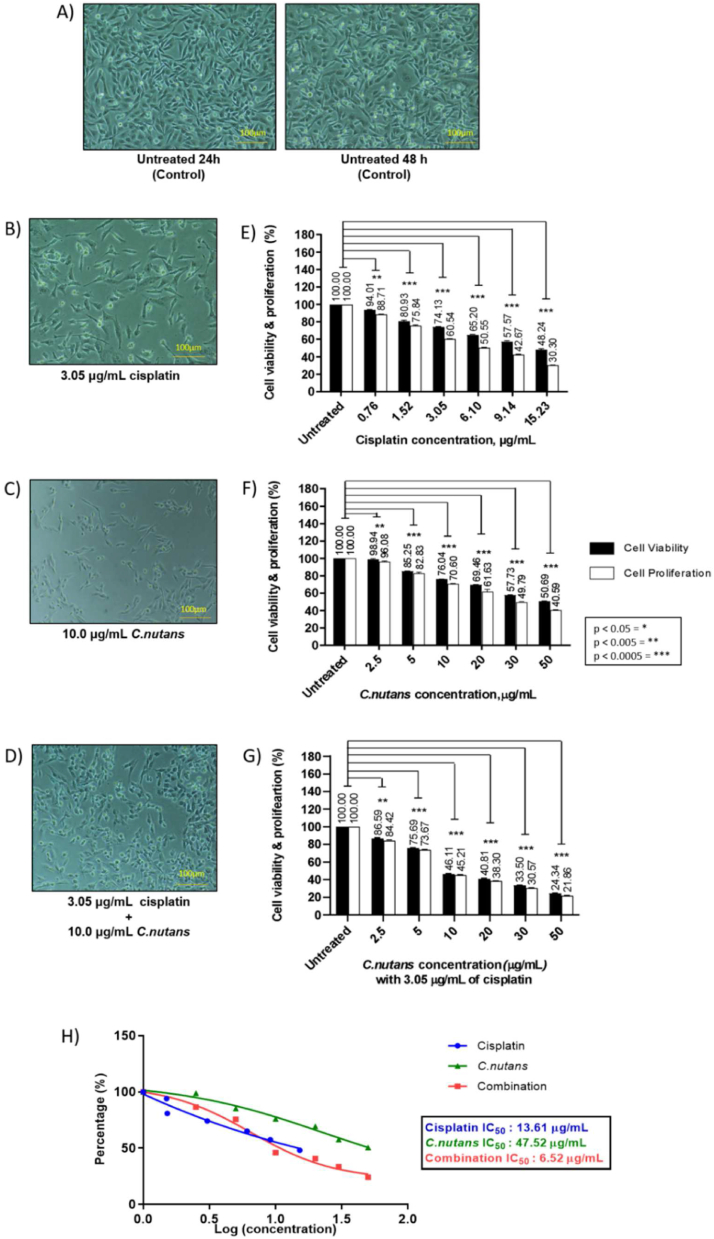
MDA-MB-231 cells (A) untreated (Control) and treated with (B) cisplatin, (C) *C. nutans,* and (D) a combination of cisplatin and *C. nutans.* The bar charts show the effect of (E) cisplatin (0.76, 1.52, 3.05, 6.10, 9.14, and 15.23 μg/mL), (F) *C. nutans* (2.5, 5, 10, 20, 30 and 50 μg/mL), (G) combined cisplatin (3.05 μg/ml)–*C. nutans* (2.5, 5, 10, 20, 30 and 50 μg/mL), and (H) dose response curve: IC_50_ concentrations achieved via cisplatin, *C. nutans* and combined cisplatin and *C. nutans* treatments.

It is evident that the combination of cisplatin and *C. nutans* on MDA-MB-231 and MDA-MB-468 cells efficaciously reduced cell viability and proliferation in a dose–response manner in comparison to the singular cisplatin and *C. nutans* treatments. Each treatment applied to MDA-MB-231 and MDA-MB-468 cells, showed similar levels of cell viability at the lowest to highest cisplatin and *C. nutans* concentrations ranging from 2 to 4% at 0.76 μg/mL of cisplatin and 2.5 μg/mL of *C. nutans*, 24–26% at 3.05 μg/mL of cisplatin and 10 μg/mL of *C. nutans*, and 49–52% at 15.23 μg/mL of cisplatin and 50 μg/mL of *C. nutans*, respectively (Figure 1B–C).

**Figure 2:**
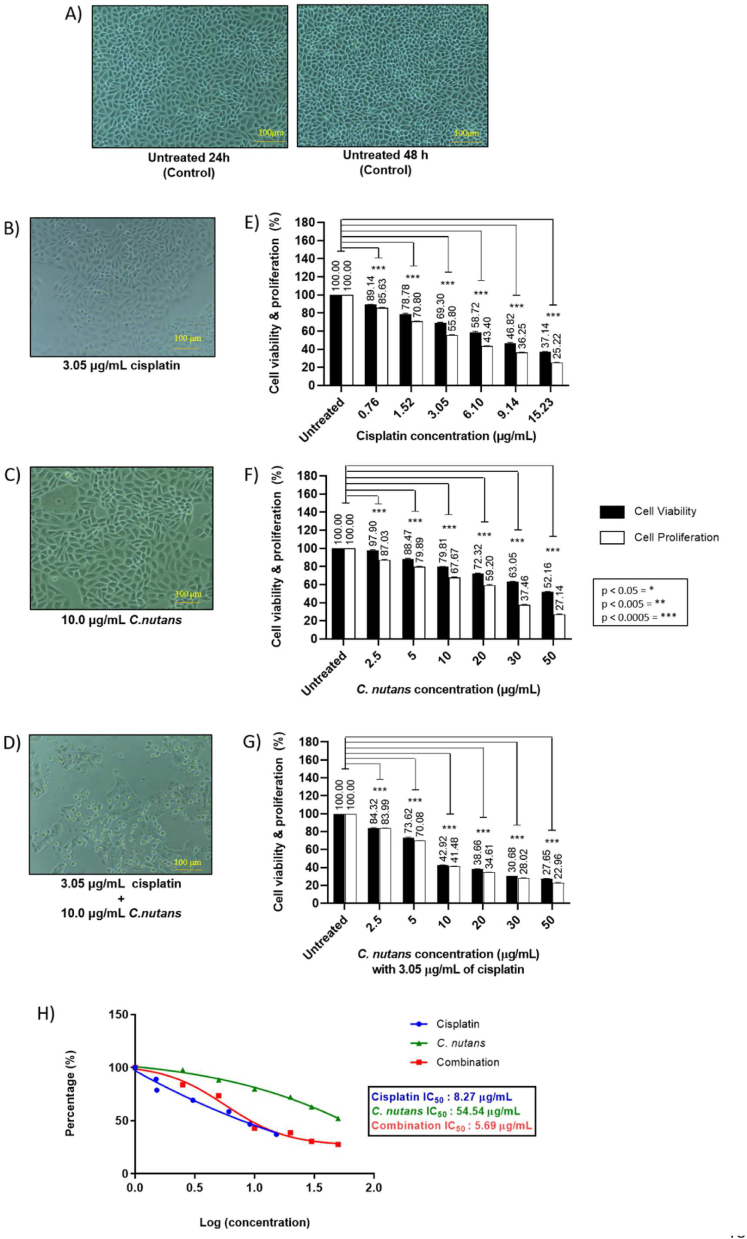
MDA-MB-468 cells (A) untreated (Control) and treated with (B) cisplatin, (C) *C. nutans* at 10 μg/mL, and (D) a combination of cisplatin and *C. nutans*. The bar charts show the effect of (E) cisplatin (0.76, 1.52, 3.05, 6.10, 9.14, and 15.23 μg/mL), (F) *C. nutans* (2.5, 5, 10, 20, 30 and 50 μg/mL), (G) combined cisplatin (3.05 μg/mL) and *C. nutans* (2.5, 5, 10, 20, 30 and 50 μg/mL), and (H) dose response curve: IC_50_ concentrations achieved via cisplatin, *C. nutans* and combined cisplatin and *C. nutans* treatments.

Similarly, the singular treatment of MDA-MB-468 cells with cisplatin and *C. nutans* showed a reduction in cell viability ranging from 2 to 11% at 0.76 μg/mL of cisplatin and 2.5 μg/mL of *C. nutans*, 20–31% at 3.05 μg/mL of cisplatin and 10 μg/mL of *C. nutans,* and 48–63% at 15.23 μg/mL of cisplatin and 50 μg/mL of *C. nutans* (Figure 2B–C). Regardless of the similar pattern of cell viability reduction seen in MDA-MB-231 cells, we observed that single cisplatin treatment exhibited a moderately higher cytotoxicity effect on MDA-MB-468 cells at all concentrations while MDA-MB-231 cells appeared marginally more sensitive towards *C. nutans* treatment in contrast to MDA-MB-468 cells. The proliferative capacity of both TNBC subtypes demonstrated a further reduction in comparison to singular treatment with cisplatin and *C. nutans* at similar concentrations. The reduction in proliferative capacity of MDA-MB-231 cells ranged between 4 and 7% at the lowest concentration, 29–39% at 3.05 μg/mL of cisplatin and 10 μg/mL of *C. nutans* treatment, and 59–70% at the highest concentrations, respectively (Figure 1B–C). In contrast, the proliferative capacity of MDA-MB-468 cells exhibited a greater inhibitory effect upon cisplatin and *C. nutans* treatment, respectively, ranging from 13 to 14.37% at 2.5 μg/mL of *C. nutans* and 0.76 μg/mL of cisplatin, 32–44.2% at 10 μg/mL of *C. nutans* and 3.05 μg/mL of cisplatin, and over 70% at 50 μg/mL of *C. nutans* and 15.23 μg/mL of cisplatin (Figure 2B–C).

In contrast to the singular treatments with cisplatin and *C. nutans*, the combined cisplatin and *C. nutans* treatment had a more potent effect on cell viability and proliferation inhibition in both TNBC subtypes. A fixed 3.05 μg/mL cisplatin treatment for 24 h prior to various *C. nutans* treatments, ranging between 10 and 50 μg/mL revealed that *C. nutans* sensitized MDA-MB-231 cells to cisplatin treatment. The combination of cisplatin and *C. nutans* treatment significantly reduced cell viability and proliferation in MDA-MB-231 cells when compared to singular *C. nutans* treatment. With a combined treatment of 3.05 μg/mL cisplatin and 2.5–50 μg/mL *C. nutans*, the percentage of reduction in cell viability and proliferation ranged between 13 and 15% for cisplatin-2.5 μg/mL *C. nutans*, 24–26% for cisplatin-5 μg/mL *C. nutans*, 45–46% for cisplatin-10 μg/mL *C. nutans*, 51–62% for cisplatin-20 μg/mL *C. nutans*, 66–69% for cisplatin-30 μg/mL *C. nutans* and 76–78% for cisplatin-50 μg/mL *C. nutans* when compared to singular 2.5 μg/mL *C. nutans* (0–4%), 5 μg/mL *C. nutans* (15–17%), 10 μg/mL *C. nutans* (24–29%), 20 μg/mL *C. nutans* (30–38%), 30 μg/mL *C. nutans* (42–50%) and 50 μg/mL *C. nutans* (49–59%), as shown in Figure 1F–G and summarized in Table 5. A similar trend was observed for MDA-MB-468 cells when treated with 3.05 μg/mL cisplatin + 2.5–50 μg/mL *C. nutans*. The percentage of reduction in cell viability and proliferation with a combined treatment of 3.05 μg/mL cisplatin and various *C. nutans* concentrations (2.5, 5, 10, 20, 30 and 50 μg/mL) were 2–13%, 12–20%, 20–32%, 28–41%, 37–63% and 48–73%, respectively, as compared to singular *C. nutans* treatment at the same concentrations (15–16%, 26–30%, 57–59%, 61–65%, 69–72% and 72–77%) respectively (Figure 2E–G; Table 5). In addition, the combined treatment also significantly reduced the IC_50_ concentration from 13.61 μg/mL (cisplatin) and 47.52 μg/mL (*C. nutans*) to 6.52 μg/mL in MDA-MB-231 cells (Figure 1H) and from 13.61 μg/mL (cisplatin) and 47.52 μg/mL (*C. nutans*) to 6.52 μg/mL in MDA-MB-231 cells and from 8.27 μg/mL (cisplatin) and 54.54 μg/mL (*C. nutans*) to 5.69 μg/mL in MDA-MB-468 cells (Figure 2H).

**Table 5:** Percentage of cell viability and proliferation reduction in MDA-MB-231 and MDA-MB-468 cells in response to treatment with cisplatin, *C. nutans* and a combination of cisplatin and *C. nutans*.

Drug concentration (μg/mL)	MDA-MB-231		MDA-MB-468	
	Viability (%)	Proliferation (%)	Viability (%)	Proliferation (%)
CP(0)	100	100	100	100
CP-0.76	94.01	88.71	89.14	85.63
CP-1.52	80.93	75.84	78.78	70.80
CP-3.05	74.3	60.54	69.30	55.80
CP-6.10	65.20	50.55	58.72	43.40
CP-9.14	57.57	42.67	46.82	36.52
CP-15.23	48.24	30.30	37.14	25.22
CN(0)	100	100	100	100
CN-2.5	98.94	96.08	97.90	87.03
CN-5.0	85.25	82.83	88.47	79.89
CN-10	76.04	70.60	79.81	67.67
CN-20	69.46	61.63	72.32	59.20
CN-30	57.73	49.79	63.05	37.46
CN-50	50.69	40.59	52.16	27.14
CP(0)-CN(0)	100	100	100	100
CP–CN-2.5	86.59	84.42	84.32	83.99
CP–CN-5	75.69	73.67	73.62	70.08
CP–CN-10	46.11	45.21	42.92	41.48
CP–CN-20	40.81	38.30	38.66	34.61
CP–CN-30	33.50	30.57	30.68	28.02
CP–CN-50	24.34	21.86	27.65	22.96

CP: cisplatin; CN: *Clinacanthus nutans*; CP–CN: cisplatin–*C. nutans*.

Prominent changes in cell morphology upon cisplatin and *C. nutans* treatments were evident in MDA-MB-231 cells (Figure 1B–D) and MDA-MB-468 cells (Figure 2B–D), respectively. MDA-MB-231 cells appeared enlarged and elongated with 3.05 μg/mL cisplatin treatment (Figure 1B; MDA-MB-231) while cells were smaller and elongated with 10 μg/mL *C. nutans* treatment (Figure 1C; MDA-MB-231). However, upon combined treatment (3.05 μg/mL cisplatin + 10–50 μg/mL *C. nutans*) (Figure 1D; MDA-MB-231), cells appeared small, shorter, and more rounded when compared to the spindled-shaped untreated cells (control) (Figure 1A; MDA-MB-231). In contrast, there was no detectable change in MDA-MB-468 cell morphology in response to 3.05 μg/mL cisplatin treatment although some cells looked slightly elongated (Figure 2B; MDA-MB-468); cells treated with 10 μg/mL *C. nutans* appeared slightly enlarged and elongated (Figure 2C; MDA-MB-468). However, upon combined treatment (3.05 μg/mL cisplatin + 10–50 μg/mL *C. nutans*), almost all remaining MDA-MB-468 cells appeared very small, rounded, and apoptotic-like when compared to the epithelial-like untreated cells (control) (Figure 2A).

**Figure 3:**
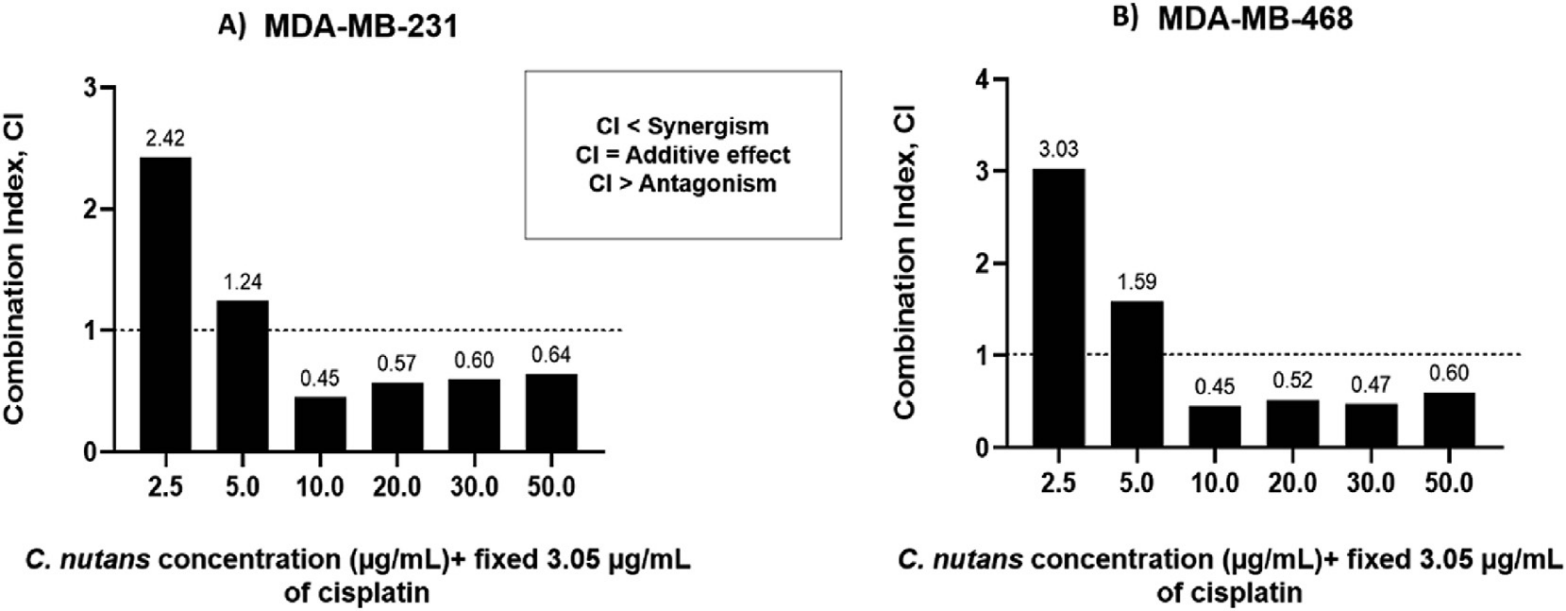
Effects of combined cisplatin and *C. nutans* treatment on (A) MDA-MB-231 cells and (B) MDA-MB-468 cells. The bar chart is plotted as a combination index (CI) against *C. nutans* concentration (2.5, 5, 10, 20, 30 and 50 μg/mL).

As a significant inhibitory effect was exhibited in both MDA-MB-231 and MDA-MB-468 cells upon combined treatment, we next investigated the drug–drug interaction of cisplatin and *C. nutans*. Interestingly, the combined treatment of cisplatin (3.05 μg/mL) and 2.5–5 μg/mL of *C. nutans* exhibited an antagonistic effect, as indicated by CI values above 1. However, the drug–drug interaction with 3.05 μg/mL of cisplatin and increasing *C. nutans* concentrations (10–50 μg/mL) showed that both agents exerted synergistic anticancer effects on MDA-MB-231 and MDA-MB-468 cells, as indicated by CI values below 1 (Figure 3).

### Combined cisplatin and *C. nutans* treatment induced apoptosis in MDA-MD-231 and MDA-MB-468 breast cancer cells

**Figure 4:**
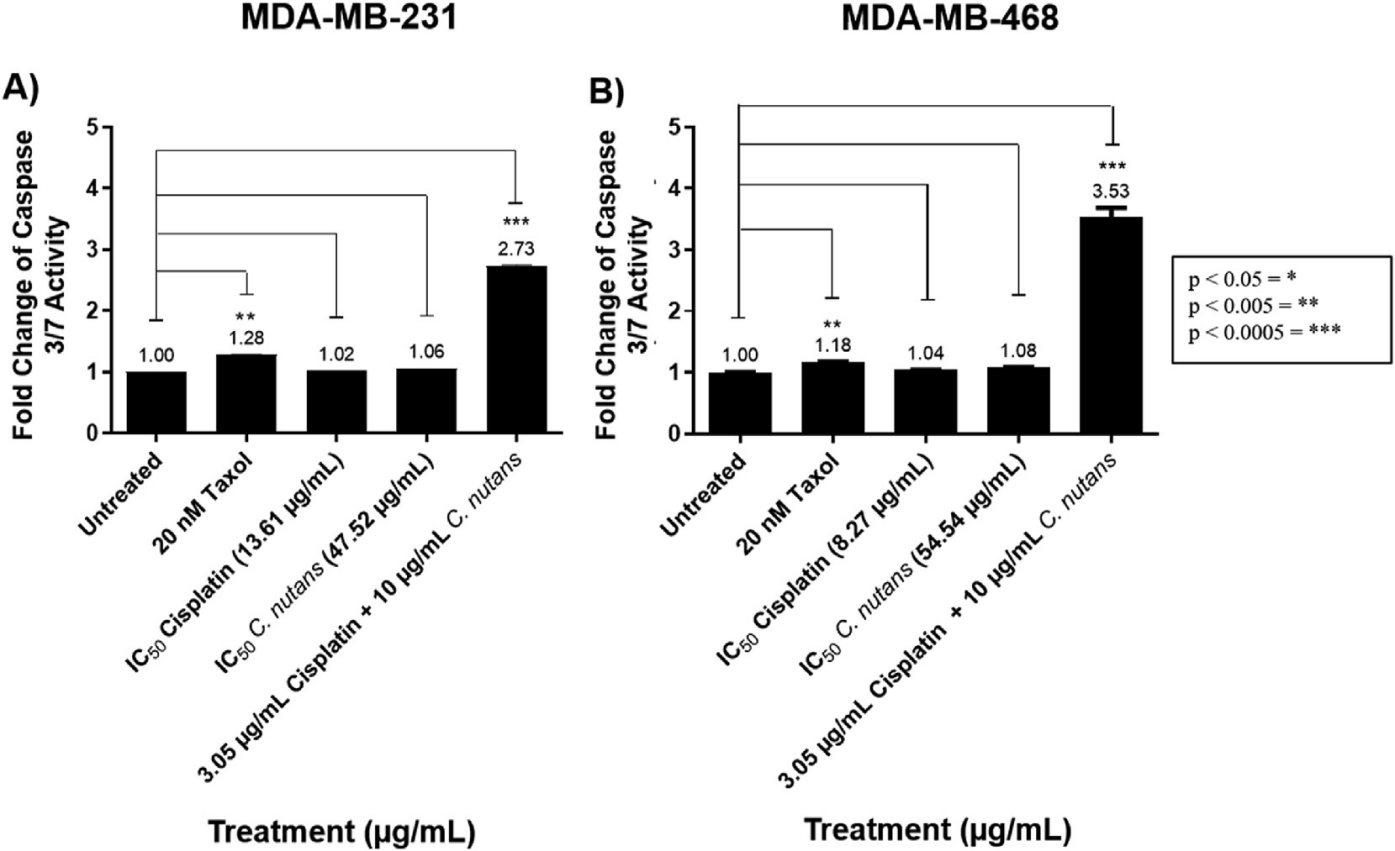
Effect of Taxol (positive control), cisplatin, *C. nutans* and combined treatment (3.05 μg/mL cisplatin + 10 μg/mL *C. nutans*) on Caspase-3/7 activity in (A) MDA-MB-231 cells and (B) MDA-MB-468 cells.

Based on the findings in Figure 1, Figure 2, it appeared that the singular cisplatin and *C. nutans* treatments potentially exerted a different mechanism of action when compared to the combined treatment. Hence, we investigated the ability of each treatment to induce apoptosis via Caspase 3/7 activity in TNBC cells represented by MDA-MB-231 and MDA-MB-468 cells (Figure 4). Figure 4 shows that the activation of Caspase-3/7 was negligible in cells treated with IC_50_ of cisplatin and *C. nutans* concentrations in contrast to treatment with 20 nM Taxol (1.28-fold and 1.18-fold) and combined treatment (3.05 μg/mL cisplatin + 10 μg/mL *C. nutans*) by 2.73-fold and 3.53-fold, respectively. This finding can be supported by the morphological changes in MDA-MB-231 and MDA-MB-468 cells upon combined treatment, which appeared unhealthy, rounded, uneven, and apoptotic-like (Figure 1, Figure 2D) in comparison to the healthy and viable untreated cells (control) (Figure 1, Figure 2A). This cells also appeared morphologically different to those receiving single cisplatin and *C. nutans* treatments (Figure 1B–C and Figure 2B–C).

### Inhibition of cell invasion by MDA-MD-231 breast cancer cells when treated with a combination of cisplatin and *C. nutans*

**Figure 5:**
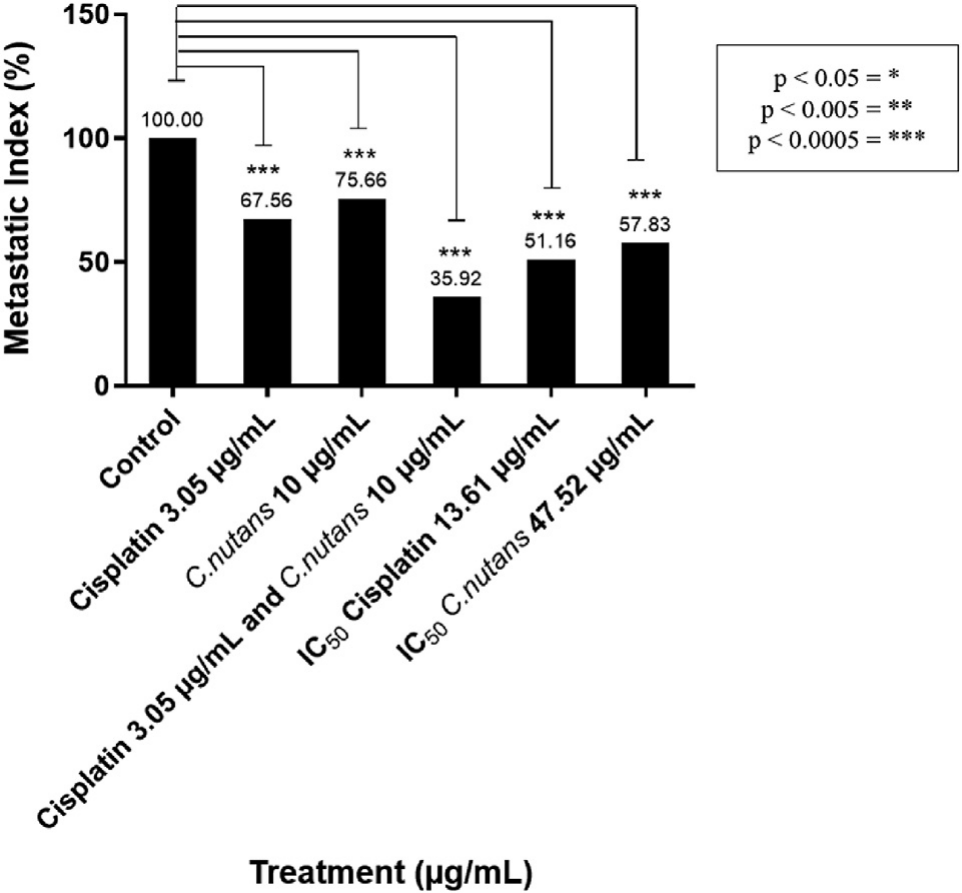
Effect of cisplatin (3.05 μg/mL; IC_50_:13.61 μg/mL), *C. nutans* (10 μg/mL; IC_50_: 47.52 μg/mL) and combined treatment (3.05 μg/mL cisplatin + 10 μg/mL *C. nutans*) on the metastatic ability of MDA-MB-231 cells.

MDA-MB-231 cells are a highly metastatic breast cancer subtype.^40^ Hence, we studied the ability of cisplatin, *C. nutans* and the combination of cisplatin and *C. nutans* to inhibit the metastatic capacity of MDA-MB-231 cells. We found that MDA-MB-231 cells exhibited reduced metastatic capacity but at different capacities; the highest level of inhibition was observed in cells treated with the combination of cisplatin and *C. nutans* when compared to moderate inhibition upon singular cisplatin and *C. nutans* treatment (Figure 5). Figure 5 shows that the lowest level of inhibition was exhibited by 10 μg/mL of *C. nutans* (24%), followed by 3.05 μg/mL of cisplatin (32%), the IC_50_ of *C. nutans* (42%), and the IC_50_ cisplatin (49%). The highest levels of inhibition in MDA-MB-231 cells were in response to combined treatment (3.05 μg/mL cisplatin + 10 μg/mL *C. nutans*) (64%).

### Differential gene regulation of KLF4, KRT18, TUBA1A and CD49f in MDA-MB-231 breast cancer cells treated with cisplatin and *C. nutans*

**Figure 6:**
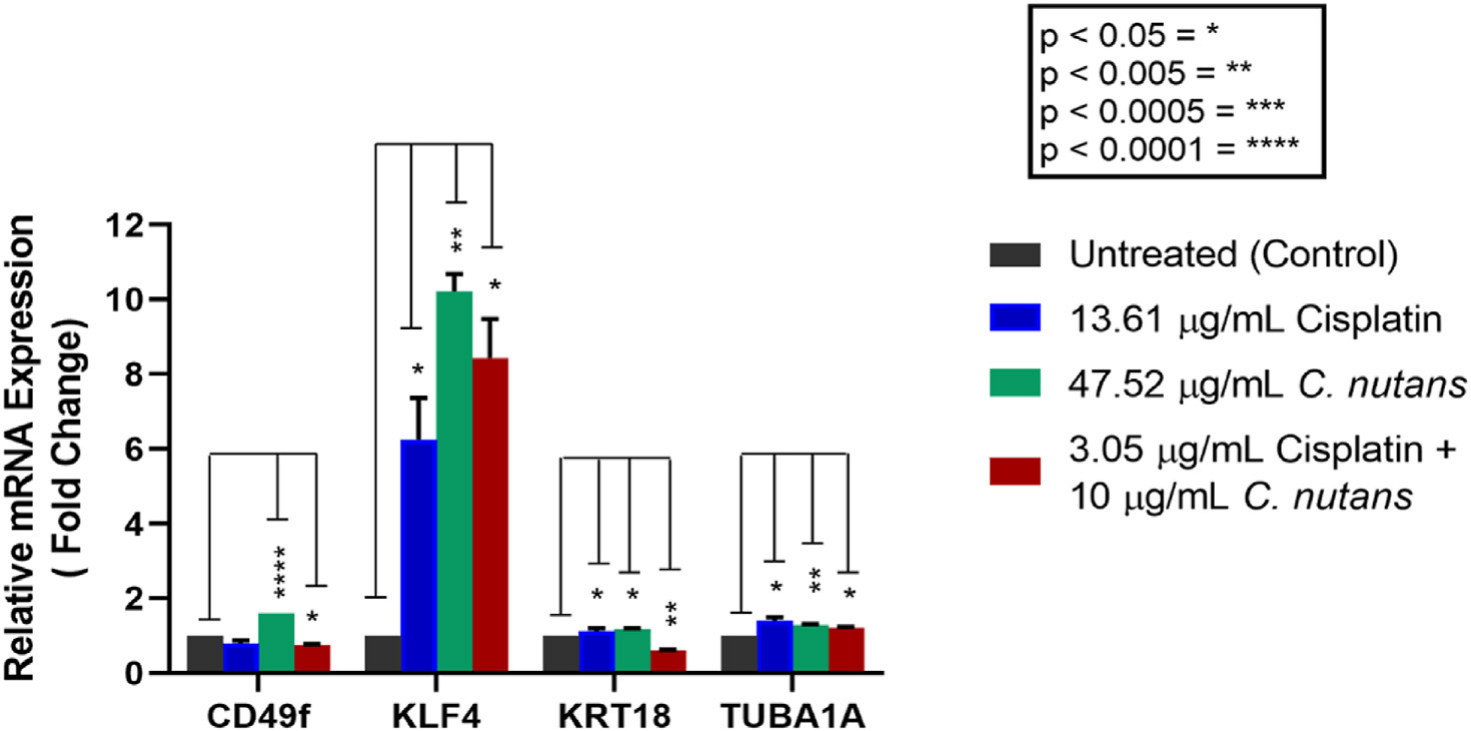
Effects of cisplatin (IC_50_: 13.61 μg/mL), *C. nutans* (IC_50_: 47.52 μg/mL) and combined treatment (cisplatin and *C. nutans* (3.05 μg/mL + 10 μg/mL)) on the mRNA expression of *CD49f*, *KLF4*, *KRT18*, and *TUBA1A* in MDA-MB-231 breast cancer cells.

To study the role of cisplatin, *C. nutans* and the combination of cisplatin and *C. nutans* on gene regulation, we next investigated the expression of several genes associated with breast cancer stem cells (integrin alpha 6, *ITGA6*, *CD49f*), metastatic markers (Kruppel-like Factor 4, *KLF4*), and differentiation markers (cytokeratin-18, *KRT18*; Tubulin alpha 1A, *TUBA1A*) (Figure 6). All treatments were carried out using the IC_50_ concentration of each drug prior to gene expression studies.

Figure 6 clearly shows that MDA-MB-231 cells expressed all of the genes studied; the expression levels of these genes were affected by treatment with cisplatin, *C. nutans* and the combination of cisplatin and *C. nutans* treatments, respectively. An obvious and significant upregulation of KLF4 mRNA expression was exhibited in MDA-MB-231 with all treatments (cisplatin, 6.256; *C. nutans*, 10.214; combination, 8.436) when compared to the expression levels of other genes. Similarly, the mRNA expression of both *KRT18* and *TUBA1A* were also up-regulated upon single cisplatin and *C. nutans* treatments; however down-regulation was observed in *KRT18* expression (39%) along with an up-regulation of *TUBA1A* expression (19%) with the combined cisplatin and *C. nutans* treatment. In contrast to the increased mRNA expression levels of *KLF4*, the expression levels of *CD49f* were down-regulated in response to cisplatin (19%) and combined cisplatin and *C. nutans* (22%) treatments. However, a significant increase in *CD49f* expression was observed in response to single *C. nutans* treatment. A down-regulation of *CD49f*, a specific marker of breast cancer stem cells, was correlated with a reduction in cancer cell stemness, reduced proliferative capacity, and the induction of differentiation.

## Discussion

Current research focuses on targeting and eliminating the resistant or CSC sub-population within a tumor, especially for difficult-to-treat cancers, including Triple Negative Breast Cancer (TNBC). This invasive form of cancer is often correlated with a poor prognosis and low survival rates due to its highly metastatic nature and the lack of targeted therapies.^41^^,^^42^ These cancers are sensitive to cisplatin treatment; however, at high doses, cisplatin often leads to severe side effects,^17^^,^^43^ in addition to the development of tumor cell resistance to cisplatin. Therefore, the use of combined therapy with cisplatin is recommended to potentially target and kill the CSCs that are responsible for the progression and metastasis of TNBCs.^44^ It is evident that various phytochemicals or plant-derived compounds can be used in combination with cisplatin and enhance the efficiency of this drug; this practice can also reduce drug concentrations and the toxicity induced by cisplatin.^11^^,^^45^

In this investigation, we demonstrated that both MDA-MB-231 (a highly metastatic and poorly differentiated cell line) and MDA-MB-468 (a metastatic and less differentiated cell line) that represent a TNBC subtype, were sensitized towards cisplatin by supplementation with an ethanolic leaf extract of *C. nutans* known to be enriched with compounds possessing anticancer properties.^31^, ^32^, ^33^, ^34^, ^35^, ^36^, ^37^, ^38^, ^39^^,^^46^ This sensitization can be proven via the achievement of a lower cisplatin IC_50_ concentration by 50% upon the treatment of addition of MDA-MB-231 cells with *C. nutans* (Figure 1H). In addition, the metastatic potential of MDA-MB-231 cells was significantly inhibited by almost 50% when treated with 3.05 μg/mL cisplatin + 10 μg/mL *C. nutans* when compared to 3.05 μg/mL cisplatin treatment. The presence of various bioactive compounds in *C. nutans* extract appears to overcome cisplatin resistance by interfering with specific signaling pathways that are crucial for CSC maintenance.^47^ These crucial signaling pathways are known to either positively regulate differentiation and apoptosis or deactivate pathways that are responsible for proliferation and metastasis. In this study, a number of bioactive compounds, including oleamide, carboxylic acid esters, and glucosides, were identified to have potential anticancer activities. Fatty acid derivatives, such as oleamide, have been shown to exert significant anticancer properties by hindering cell proliferation in various cell lines,^34^^,^^48^, ^49^, ^50^ including MDA-MB-231 cells.^51^ Carboxylic acid esters that are present in plants, including *C. nutans*, play an important role as bioactive compounds in various cancer cell lines. The presence of carboxylic acid ester groups in synthesized cisplatin–acridine hybrids has been associated with the significant inhibition of cell proliferation in ovarian and breast cancer cells.^52^, ^53^, ^54^ The third most abundantly present bioactive compound of *C. nutans*, glycosides, are commonly found in many plants and have demonstrated strong cytotoxic effects in various cancer cell lines.^55^^,^^56^ The anticancer of *C. nutans* was facilitated by numerous mechanisms. For example, flavonoid glycosides exerted obvious anticancer activity in HeLa cells via an apoptosis mechanism.^57^ In addition, paeoniflorin, a representative of glycoside, showed antitumor effects on a diverse range of tumors, both *in vivo* and *in vitro*, including breast cancer via the induction of tumor cell apoptosis in addition to the inhibition of proliferation, tumor invasion and metastasis.^58^ It is most likely that the abundance of oleamide, carboxylic acid esters, and glucosides, in *C. nutans* extracts is responsible for the enhanced inhibitory effects in MDA-MB-231 and MDA-MB-468 cells. However, it is important to further investigate each bioactive compound present in *C. nutans* extracts and correlate these with anticancer properties via the treatment of MDA-MB-231 and MDA-MB-468 cells with a combination of cisplatin and *C. nutans.*

When correlating the ability to inhibit cell viability and proliferation of MDA-MB-231 and MDA-MB-468 cells, it can be postulated that both cisplatin and *C. nutans* treatments were able to limit the proliferative capacity, although cells were viable post-treatment (Figure 1D–E and Figure 2D–E). This may suggest that singular cisplatin and *C. nutans* may have induced the differentiation of MDA-MB-231 and MDA-MB-468 cells based on morphological changes (enlarged and elongated cells; thinner/smaller and elongated cells), as seen in Figure 1B–C and Figure 2B–C, respectively. Cisplatin is commonly known to interfere with DNA activity in tumors by the formation of adducts (inter-strand or intra-strand crosslinks) and consequently preventing the DNA repair process, thus leading to apoptosis.^59^^,^^60^ However, it appears that cisplatin may have a different mode of action in response to resistant CSCs. A previous study showed that cisplatin is able to induce differentiation in TNBCs enriched with CSCs^1^ but lacks therapeutic efficacy as a single anticancer agent.^11^^,^^44^ This differentiation by cisplatin is presumed to be caused by the binding of cisplatin compounds to other target molecules apart from nuclear DNA, including some peptides, cellular proteins, and RNAs.^16^^,^^61^^,^^62^ The binding of cisplatin to these targets could also lead to epigenetic modifications by switching on and/or off the expression of specific epigenetic biomarkers related to stemness and differentiation.^63^^,^^64^ In contrast, as yet, no researchers have correlated *C. nutans* and the differentiation of cancer cells. Nevertheless, studies have shown that oleamide, a fatty acid derivative, plays important roles as a gap junction modulator and anti-angiogenic agent, thus resulting in reduced metastases and invasion.^51^^,^^65^ Some gap junction proteins assist in intracellular communication between tumor cells and causes these cells to go undetected by the immune system, thus leading to cancer metastases and progression.^66^, ^67^, ^68^ Previous studies have shown that the gap junction proteins Connexin-26 (Cx26) and Connexin-43 (Cx43) are often upregulated and downregulated, respectively, in the CSC population in TNBCs which are responsible for driving self-renewal.^47^^,^^66^^,^^68^^,^^69^ Oleamide is known to modulate the function of gap junction proteins via negative and positive regulation by interrupting the intracellular communication between tumor cells. These regulatory events consequently lead to the structural inhibition of gap junction channels^51^^,^^66^ and the sensitization of CSCs to certain chemotherapeutic drugs, such as cisplatin by improving its uptake.^68^^,^^70^ However, some studies have contradicted these findings by observing that oleamide increased survival and proliferation upon combined treatment in manner that depended on the type of cancer cell and the anticancer agent used together with oleamide.^71^^,^^72^ Furthermore, butanedioic acid, a carboxylic acid ester, is known to be a metabolic suppressor and can lead to reduced proliferation and stemness.^73^^,^^74^ In short, the ethanolic extract of *C. nutans*, which is enriched with fatty acids, carboxylic acid esters (long chain fatty acids), glycosides, and their derivatives, are presumed to be the major contributing bioactive compounds exerting anti-cancerous activities through multiple pathways.^34^^,^^75^, ^76^, ^77^, ^78^

In comparison to single treatments, the combined treatment of cisplatin and *C. nutans* on MDA-MB-231 and MDA-MB-468 cells significantly promoted a synergistic anticancer effect with increasing *C. nutans* concentration. The combined treatment at a lower *C. nutans* concentration (fixed at 3.05 ug/mL of cisplatin and *C. nutans* at 2.5 and 5 ug/mL) exerted less cytotoxic effects in both of the cell lines tested. It is presumed that at low doses, *C. nutans* is not capable of inducing a significant inhibitory effect but reacts in an antagonistic manner.^79^^,^^80^ However, a clear shift from antagonistic to synergistic effects was observed with increasing concentrations of *C. nutans*. The presence of a high fatty acid content in the extract of *C. nutans* leaves that are lipophilic in nature could be a contributing factor in increasing the bioavailability and uptake of cisplatin into tumor cells in addition to its own anticancer properties.^34^^,^^81^ Although cisplatin is known for its potent anticancer activity on various solid tumors, the cisplatin-plasma membrane interaction is often correlated with poor uptake and resistance.^82^^,^^83^ Consequently, a higher dosage may be required; however, this would, in turn, cause adverse side effects in patients. The presence of fatty acids in the form of oleamide, as the major bioactive compound in *C. nutans* leaf extract, in combination with cisplatin, appears to complement the uptake of cisplatin more efficiently into tumor cells and in addition interacts with cisplatin to produce synergistic anticancer effects.

In addition, the combined treatment also significantly reduced the cell viability and proliferation by more than 70%; the remaining cells were partially dead or undergoing early apoptosis (Figure 1, Figure 2D and G). Figure 4 confirms this claim as a significant induction of caspase 3/7 activity was exhibited in the remaining cisplatin–*C. nutans* treated cells when compared to Taxol (the positive control) as well as cells treated with singular cisplatin and *C. nutans*. The seemingly reduced cell viability and proliferation at a similar percentage in the combined treatment also suggest that the reduction is due to cell death, which correlates well with the activation of caspase-3/7 activity (Figure 1, Figure 2D, G and 4). This interaction between the major bioactive compounds in *C. nutans* and cisplatin sensitized resistant TNBC cells to cisplatin and ultimately initiated apoptosis in TNBC cells. The negligible apoptotic induction in single cisplatin and *C. nutans* treated cells in contrast, may suggest that the remaining cells did not undergo apoptosis but possibly were differentiated instead; this could have led to the compromised stemness of cancer cells, along with limited proliferative and metastatic capacity^84^ in MDA-MB-231 cells, as demonstrated by the reduced metastatic index and the downregulation and upregulation of breast CSCs and differentiation markers, respectively (Figure 5, Figure 6).

Cisplatin treated MDA-MB-231 cells exhibited a downregulation in the mRNA expression of *CD49f* (a breast CSC marker) and an upregulation of *TUBA1A* and *KRT18* (neural and luminal epithelial differentiation markers). With regards to *C. nutans* treated MDA-MB-231 cells, *CD49f* mRNA expression was upregulated along with *KRT18* and *TUBA1A*. This finding may suggest that *C. nutans* did not interfere with the expression of *CD49f* at the gene level. However, it would be interesting to investigate the expression of CD49f protein as *C. nutans* could induce epigenetic modification of CD49f expression at the post-translational level. The findings from our gene expression study support the previous claims made based on Figure 1, Figure 2, Figure 3 in which the single treatments potentially induced differentiation. In addition to *CD49f*, *KRT18* and *TUBA1A,* the expression of *KLF4* was significantly higher than the expression levels of other selected genes (*CD49f*, *KRT18*, and *TUBA1A*) upon single cisplatin and *C. nutans* as well as combined cisplatin and *C. nutans* treatments. KLF4 is an embryonic stem cell (ESC) marker and a transcription factor (TF) that is expressed at high levels by most breast cancer cell types.^85^^,^^86^ KLF4 acts as a transcriptional factor responsible for epithelial cell proliferation and differentiation.^87^ Generally, KLF4 has been considered as a negative cell cycle regulator, monitoring multiple genes to promote and inhibit proliferation.^87^ KLF4 has been recognized as one of the “pluripotency genes” due to its ability of induce pluripotent stem cells.^87^^,^^88^ KLF4 is able to reprogram normal somatic cells into stem-like cells to maintain its ability to self-renew and prevent differentiation.^87^^,^^89^ Despite KLF4 being an early-stage ESC and a TF that aids in the maintenance of the embryonic stage in cancer cells, the expression of KLF4 in TNBCs may act as a gene that suppresses tumor growth by targeting CSCs. This finding can be supported by a few recent findings which claim that KLF4 can have dual functions in which it either acts as a potent oncogenic activator or a tumor suppressor, as determined by the type of cancer and its microenvironment.^3^^,^^88^^,^^90^ Previous studies showed that high levels of KLF4 expression in TNBC patients correlated with better overall survival and cancer-free survival rate^42^ and also correlated with the sensitization of cancer cells to cisplatin and paclitaxel treatment.^91^

An earlier study by Zhang et al., in 2010 showed that KLF4 interacts with NANOG, another TF, to prevent ESC differentiation; however, the knockdown of NANOG resulted in differentiation.^92^ This argument can be well correlated with MDA-MB-231 cells, as these invasive TNBC cells are enriched with CSCs but lack NANOG gene expression.^93^ Therefore, the lack of NANOG expression by MDA-MB-231 cells may have switched the oncogenic KLF4 to tumor suppressor mode, which consequently induced differentiation upon cisplatin and *C. nutans* treatment. With regards to *KLF4* gene up-regulation and the induction of differentiation, previous studies have claimed that KLF4 specifically induces the differentiation of epithelial cell origin^92^^,^^94^ which again may explain the correlation of KLF4 and the differentiation induction of MDA-MB-231 cells which are epithelial in origin.^95^ In comparison to the single cisplatin and *C. nutans* treatments, the combined treatment downregulated *CD49f* and *KRT18* expression; this may have arisen as the number of apoptotic cells increased.

Collectively, these findings suggest that the synergistic effect of combined cisplatin–*C. nutans* treatment is closely related to the interactions of oleamide, the major bioactive compound in *C. nutans*. Oleamide modulates gap junction proteins and may help to sensitize MDA-MB-231 and MDA-MB-468 cells by upregulating Cx43 which has been shown to increase the uptake of cisplatin^70^ and promote apoptosis while downregulating Cx26 which is responsible for metastasis and cancer progression.^68^^,^^96^ This is interesting because gap junction communication plays an important role in calcium (Ca^2+^) signaling whereas cisplatin uptake, along with its cytotoxicity and chemosensitivity, are dependent on the cytosolic calcium concentration.^97^ This claim can be supported by the lower cisplatin and IC_50_ concentrations required to exert significant anticancer activity in MDA-MB-231 and MDA-MB-468 cells apart from apoptotic induction and the limited metastatic ability via the upregulation of differentiation and the downregulation of CSC makers. However, the mechanisms by which oleamide can induce connexins to mediate cisplatin chemosensitivity remain unclear and need to be investigated further.

**Figure 7:**
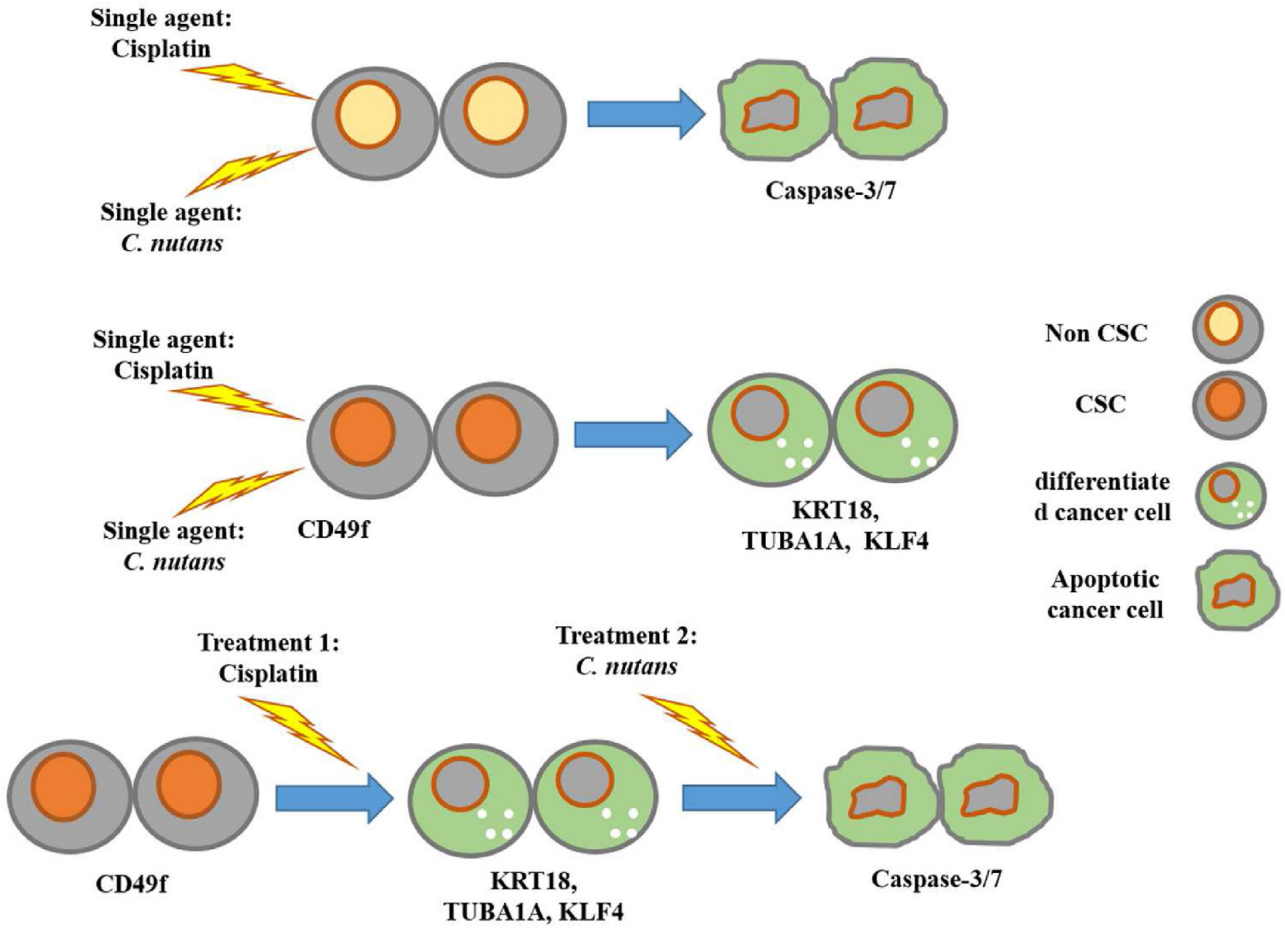
The possible mechanism of action exerted by the combined cisplatin and *C. nutans* treatment on TNBC cells. Cisplatin induces breast cancer stem cell differentiation, making them sensitive to *C. nutans* treatment and consequently undergo apoptosis. The combined cisplatin-phytochemical combination may be beneficial in the treatment and management of TNBCs.

In summary, the combination of cisplatin and phytochemicals, such as *C. nutans*, could be a potential therapeutic option for treating and managing TNBCs and possibly other types of cancers that are enriched with CSCs. Figure 7 shows a proposed model based on our findings, which highlights the possible mechanism of action exerted by the combined treatment of TNBC cells with cisplatin and *C. nutans*.

## Conclusion

To conclude, both cisplatin and *C. nutans* were found to be potent anticancer agents and induced the differentiation of tumor cells as a single anticancer agent while promoting cell death via apoptosis upon combined cisplatin and *C. nutans* treatment which targeted the more differentiated MDA-MB-231 and MDA-MB-468 cells. Gene expression studies also revealed that cisplatin and *C. nutans* differentially regulated specific genes in MDA-MB-231 TNBC cells. Although the exact mechanism of the cisplatin and *C. nutans* combination remains unclear, we showed that each of the anticancer agents may possess multiple mechanisms of action. We also demonstrated that *C. nutans* leave extract is enriched with fatty acids, which may have acted as a drug carrier for cisplatin uptake into tumor cells more efficiently in addition to the synergistic interactions of cisplatin and *C. nutans*, thus leading to apoptotic induction. Further investigations are now required to gain a better understanding of how the cisplatin and *C. nutans* combination interacts with s as well as other solid tumor microenvironments.

## Source of funding

This research was funded by the Fundamental Research Grant Scheme (FRGS) by the 10.13039/501100002385Ministry of Higher Education (MoHE), Malaysia (Reference Code. FRGS/1/2015/SKK08/UTM//1).

## Conflict of interest

The authors declare that there are no conflicts of interest.

## Ethical approval

No ethical approval was required as this study did not involve human participants or laboratory animals.

## Authors contributions

Conceptualization: PP. Methodology: PP, VL and PM. Validation: PP and KJ. Formal analysis: NFAAB and YZL. Investigation: NFAAB and YZL. Resources: PP, KJ and VL. Data curation: NFAAB and PP. Writing – Original Draft: NFAAB and YZL. Writing – Review & Editing: FH, VL, PM, KJ and PP. Visualization: NFAAB. Supervision: PP and KJ. Project administration: PP. Funding acquisition: PP. All authors have critically reviewed and approved the final draft and are responsible for the content and similarity index of the manuscript.

## Acknowledgment

The authors express their gratitude towards the facilities, scientific and technical assistance provided by laboratory members and support staff from the Cancer Research and Animal Cell Culture Laboratories as well as Dr. Faezah Mohd. Salleh from the Plant Biotechnology Laboratory, Department of Biosciences, Universiti Teknologi Malaysia in completing this work.

## Data availability

The data are available in Dryad at https://datadryad.org/stash/share/G_7l1pgLkvTRPv_ppN9UTHDRxOOXifAEdk8pdZFLb8Y.
